# CD11c^+^ macrophages are proangiogenic and necessary for experimental choroidal neovascularization

**DOI:** 10.1172/jci.insight.168142

**Published:** 2023-04-10

**Authors:** Steven Droho, Amrita Rajesh, Carla M. Cuda, Harris Perlman, Jeremy A. Lavine

**Affiliations:** 1 Department of Ophthalmology and; 2 Division of Rheumatology, Department of Medicine, Feinberg School of Medicine, Northwestern University, Chicago, Illinois, USA.

**Keywords:** Inflammation, Ophthalmology, Macrophages, Retinopathy

## Abstract

Patients with neovascular AMD (nAMD) suffer vision loss from destructive angiogenesis, termed choroidal neovascularization (CNV). Macrophages are found in CNV lesions from patients with nAMD. Additionally, *Ccr2^–/–^* mice, which lack classical monocyte–derived macrophages, show reduced CNV size. However, macrophages are highly diverse cells that can perform multiple functions. We performed single-cell RNA-Seq on immune cells from WT and *Ccr2^–/–^* eyes to uncover macrophage heterogeneity during the laser-induced CNV mouse model of nAMD. We identified 12 macrophage clusters, including Spp1^+^ macrophages. Spp1^+^ macrophages were enriched from WT lasered eyes and expressed a proangiogenic transcriptome via multiple pathways, including vascular endothelial growth factor signaling, endothelial cell sprouting, cytokine signaling, and fibrosis. Additionally, Spp1^+^ macrophages expressed the marker CD11c, and CD11c^+^ macrophages were increased by laser and present in CNV lesions. Finally, CD11c^+^ macrophage depletion reduced CNV size by 40%. These findings broaden our understanding of ocular macrophage heterogeneity and implicate CD11c^+^ macrophages as potential therapeutic targets for treatment-resistant patients with nAMD.

## Graphical abstract

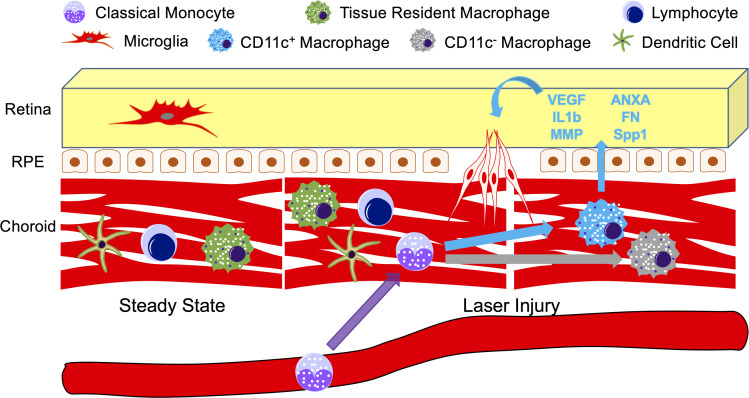

## Introduction

Neovascular age-related macular degeneration (nAMD) is the most common cause of vision loss in the developed world. Early AMD is characterized by the accumulation of inflammatory lipoprotein deposits (drusen bodies) underneath the retina that are composed of complement factors and components (1). Drusen bodies create a chronic, inflammatory microenvironment, resulting in chemotaxis of innate immune cells like macrophages (Macs) (2). This degenerative and inflammatory microenvironment underneath the retina can lead to destructive angiogenesis termed choroidal neovascularization (CNV), which ultimately causes vision loss. Current therapy includes frequent intravitreal anti–vascular endothelial growth factor (VEGF) injections (3, 4). However, 15% of patients lose vision despite aggressive monthly therapy (5). This group includes more than 2.5 million people today and is estimated to comprise 4.3 million people by the year 2040 (6). For these millions of patients, an unmet need exists for alternative therapies.

Macs are a potential therapeutic target for anti-VEGF–resistant patients with nAMD. Multiple complement genes are associated with AMD (7–9), drusen bodies include complement proteins (1), and Macs express both complement proteins and their receptors (10). Furthermore, Macs are detectable in surgically excised CNV membranes from patients with nAMD (11). These human data suggest that Macs may be key players in the genome-wide associations between AMD and the complement pathway. In the laser-induced CNV mouse model of nAMD, global Mac depletion with clodronated liposomes reduces CNV area (12, 13). Additionally, inhibition of classical monocyte (CMo) recruitment to tissue using *Ccr2^–/–^* mice reduces CNV area equivalently to clodronated liposomes (14–16). In agreement, CCR2^+^ monocytes are increased in patients with nAMD (17, 18). These data suggest that drusen bodies drive inflammation via complement in the subretinal space, leading to recruitment of CMos and differentiation of monocytes into Macs. These CMo-derived Macs (MDMs) then stimulate destructive angiogenesis during CNV.

Macs are highly heterogeneous cells in vivo that can display diverse transcriptional signatures (19). Within ophthalmology, however, most studies still rely on the M1/M2 paradigm. Analysis of M1 versus M2 markers identify more M2-like (CD163^+^CCL22^+^) Macs in excised CNV membranes from patients (11, 20, 21). Likewise, a proangiogenic Mac phenotype (Arg1^hi^Cd163^hi^), similar to the M2 paradigm (11), is detected within CNV membranes in aged mice. However, these studies only examine a few markers and do not fully capture the true heterogeneity that Macs can display. Therefore, Mac heterogeneity exists, but there is a significant scientific gap in knowledge regarding how Mac heterogeneity influences CNV.

To further our understanding of Mac origin and heterogeneity, we performed single-cell RNA-Seq (scRNA-Seq) on immune cells from WT and *Ccr2^–/–^* eyes. We identified 12 Mac populations, including a population that expressed many proangiogenic genes, was enriched from WT lasered eyes and absent from lasered *Ccr2^–/–^* eyes, and expressed the cell surface marker CD11c. Using multiparameter flow cytometry and immunofluorescence (IF) imaging, we confirmed that CD11c^+^ Macs are significantly increased in the choroid and retina after laser injury, and they are present within CNV lesions. Finally, we created CD11c^+^ Mac–depleting mice by breeding *CD11c^Cre-GFP^* and *Csf1r^LoxP-stop-LoxP-DTR-mCherry^* mice (CD11c^+^Mac^iDTR^). Diphtheria toxin (DT) treatment caused a significant reduction in CD11c^+^ Macs and laser-induced CNV area in CD11c^+^Mac^iDTR^ mice. These data suggest that CD11c^+^ Macs are proangiogenic, necessary for CNV, and could be a potential treatment for anti-VEGF–resistant patients with nAMD.

## Results

**Figure 1:**
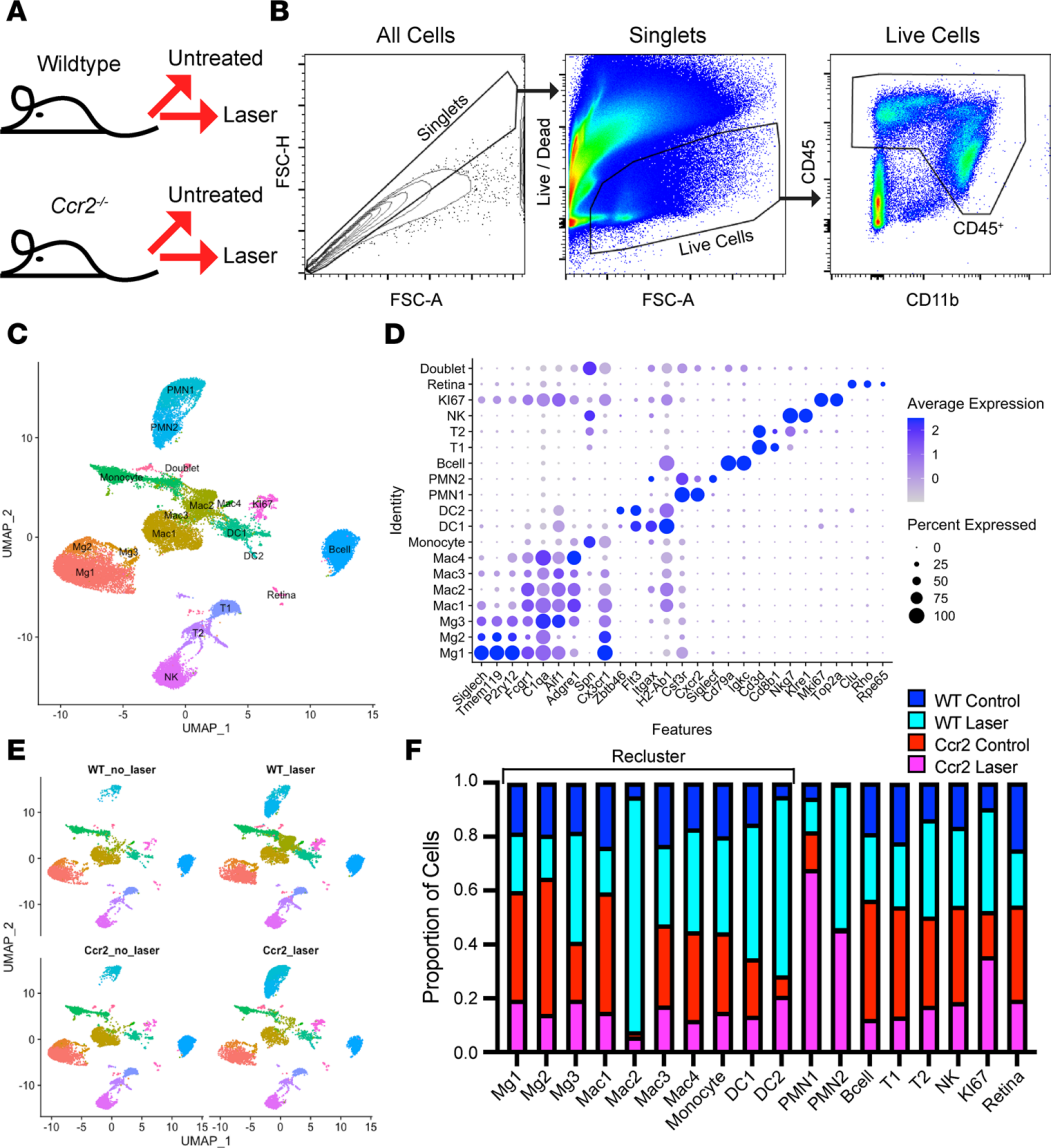
Single-cell identification of CD45^+^ immune cells. (**A**) Schematic of experimental groups (*n* = 9–15 mice per group). (**B**) Flow cytometry gating strategy for Live and Singlet CD45^+^ immune cells from whole mouse eyes for scRNA-Seq. (**C**) UMAP dimension plot. (**D**) Dot plot of marker genes for each cell type. (**E**) UMAP dimension plot split by experimental group. (**F**) Proportion of cells derived from each experimental group. Black bracket indicates cell types used for reclustering in Figure 2.

In order to determine the identity and function of each Mac subset in an unbiased manner during laser-induced CNV, we performed scRNA-Seq. Female 10- to 12-week-old C57BL6/J (WT) and *Ccr2^–/–^* mice underwent no treatment or laser injury (Figure 1A, n = 9–15 mice per group). Since *Ccr2^–/–^* mice show reduced monocyte recruitment to tissue and decreased CNV area (14), our goal was to identify a group of Macs that expressed proangiogenic genes, were increased by laser in WT mice, and were absent in both unlasered and lasered *Ccr2^–/–^* mice. On Day 3, the peak of Mac recruitment (15), eyes were enucleated and dissected to remove orbital tissue, conjunctiva, optic nerve, and extraocular muscles. Systemic perfusion was not performed because it previously had no effect upon ocular immune cell numbers in control or after laser injury using multiparameter flow cytometry (22). The whole eye, including cornea, iris, ciliary body, lens, vitreous, retina, choroid, and sclera, was digested into a single-cell suspension, and CD45^+^ cells were isolated by FACS for scRNA-Seq (Figure 1B). The whole eye was chosen to increase immune cell yield to target 10,000 sequenced cells without needing a large number of animals. After Cell Ranger alignment to the genome and exclusion of empty droplets, 34,215 cells were sequenced with an average 36,890 reads per cell. The data were loaded into Seurat v3 (23), the 4 objects were merged, and quality control metrics were performed to remove doublets and dead cells. Principal component analysis (PCA) was performed, followed by cell clustering, and visualization by uniform manifold approximation and projection (UMAP; Figure 1C). We identified Macs 1–4 (Mac1–4) as *Fcgr1*/CD64^+^*C1qa*^+^*Aif1*/IBA1^+^*Adgre1*/F4/80^+^ cells, and microglia 1–3 (Mg1–3) as uniquely *Siglech*^+^*Tmem119*^+^*P2ry12*^+^ Macs (Figure 1D). Monocytes were *Fcgr1*^–^*Spn*/CD43^+^*Cx3cr1*^+^ cells. Two DCs (DC1–2) were found that expressed *Flt3*, *Zbtb46*, *Itgax*/CD11c, and *H2-Ab1*/MHCII but lacked *Fcgr1* expression. We also identified *Cxcr2*^+^*Csf3r*^+^ polymorphonuclear cells (PMN1–2), *Cd79a*^+^*Igkc*^+^ B cells, *Cd3d*^+^*Cd8b1^+^* T cells (T1–2), *Nkg7*^+^*Klre1*^+^ NK cells, *Mki67*^+^*Top2a*^+^ proliferating cells (KI67), few contaminating retinal cells (*Clu*^+^ [muller glia], *Rho*^+^ [rods], *Rpe65*^+^ [retinal pigment epithelium]), and rare doublets (Figure 1D). The proportional contribution from the 4 experimental groups to each cluster is shown in Figure 1, E and F, and the proportional cells that each cluster contributes to the 4 experimental groups is shown in Supplemental Table 1 (supplemental material available online with this article; https://doi.org/10.1172/jci.insight.168142DS1). Since our goal was to comprehensively analyze Mac heterogeneity during laser-induced CNV, we created a subset of mononuclear phagocytes including Mg1–3, Mac1–4, Monocytes, and DC1–2, and reclustered these cells (Figure 1F). We included monocytes and DCs in this step because distinguishing Macs from monocytes and DCs can be challenging; thus, we wanted to make this distinction at a higher resolution to avoid the loss of Macs from our analysis.

**Figure 2:**
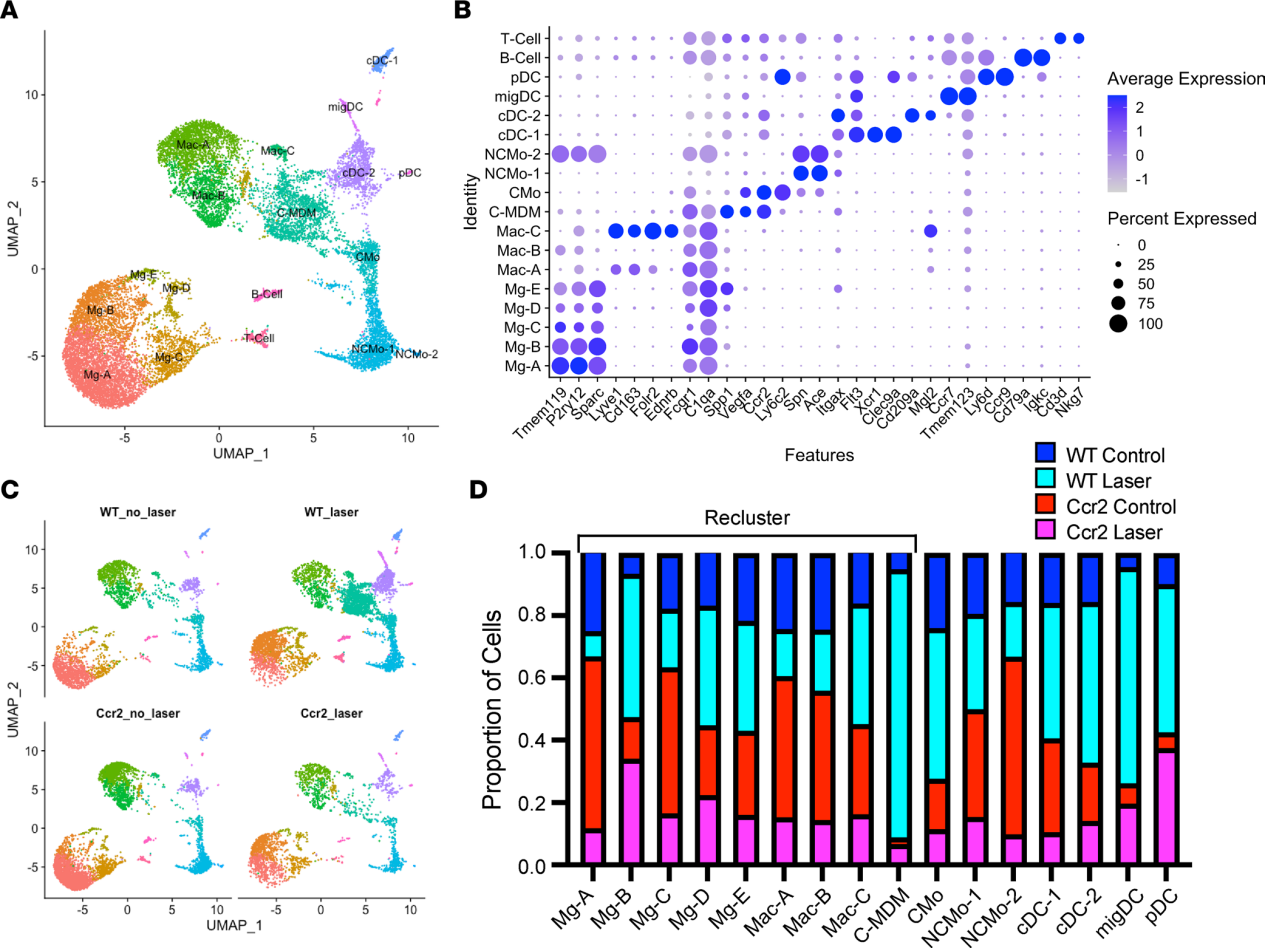
Single-cell identification of ocular mononuclear phagocytes. (**A**) UMAP dimension plot. (**B**) Dot plot of marker genes for each cell type. (**C**) UMAP dimension plot split by experimental group. (**D**) Proportion of cells derived from each experimental group. Black bracket indicates cell types used for reclustering in Figure 3.

Reclustering of mononuclear phagocytes was performed by rescaling the data, repeating PCA, cell clustering, and visualizing by UMAP (Figure 2A). We identified 5 *Sparc*^+^*Tmem119*^+^*P2ry12*^+^ Mg clusters (Mg-A–Mg-E) and 4 Mac *Fcgr1*^+^*C1qa*^+^ subsets (Mac-A–Mac-C and classical MDM [C-MDM]; Figure 2B). One Mac cluster expressed high levels of *Vegfa* and *Ccr2*, suggesting that this cluster included proangiogenic C-MDM (Figure 2B). Furthermore, C-MDM was predominantly derived from WT laser-treated mice, supporting that this cluster is derived from CMos (Figure 2, C and D). We also found *Ccr2*^+^*Ly6c2*^+^ CMos and *Spn*^+^ *Ace*^+^ non-CMos (NCMo-1; Figure 2B). Four subtypes of DCs included *Xcr1*^+^*Clec9a*^+^ classical DC type 1 (cDC-1), *Cd209a*^+^*Mgl2*^+^ cDC-2, *Ccr7*^+^ *Tmem123*^+^ migratory DC (migDC), and *Ly6d*^+^ *Cc9*^+^ plasmacytoid DCs (pDC) (24, 25). Finally, 3 rare doublet populations remained, including B cells, T cells, and non-CMos with Mg (NCMo-2). The proportional contribution from the 4 experimental groups to each cluster is shown in Figure 2D, and the proportion of cells that each cluster contributes to the 4 experimental groups is shown in Supplemental Table 2. To create our final Mac subset, we reclustered the Mg (Mg-A–Mg-E) and Mac (Mac-A–Mac-C and C-MDM) populations (Figure 2D).

**Figure 3:**
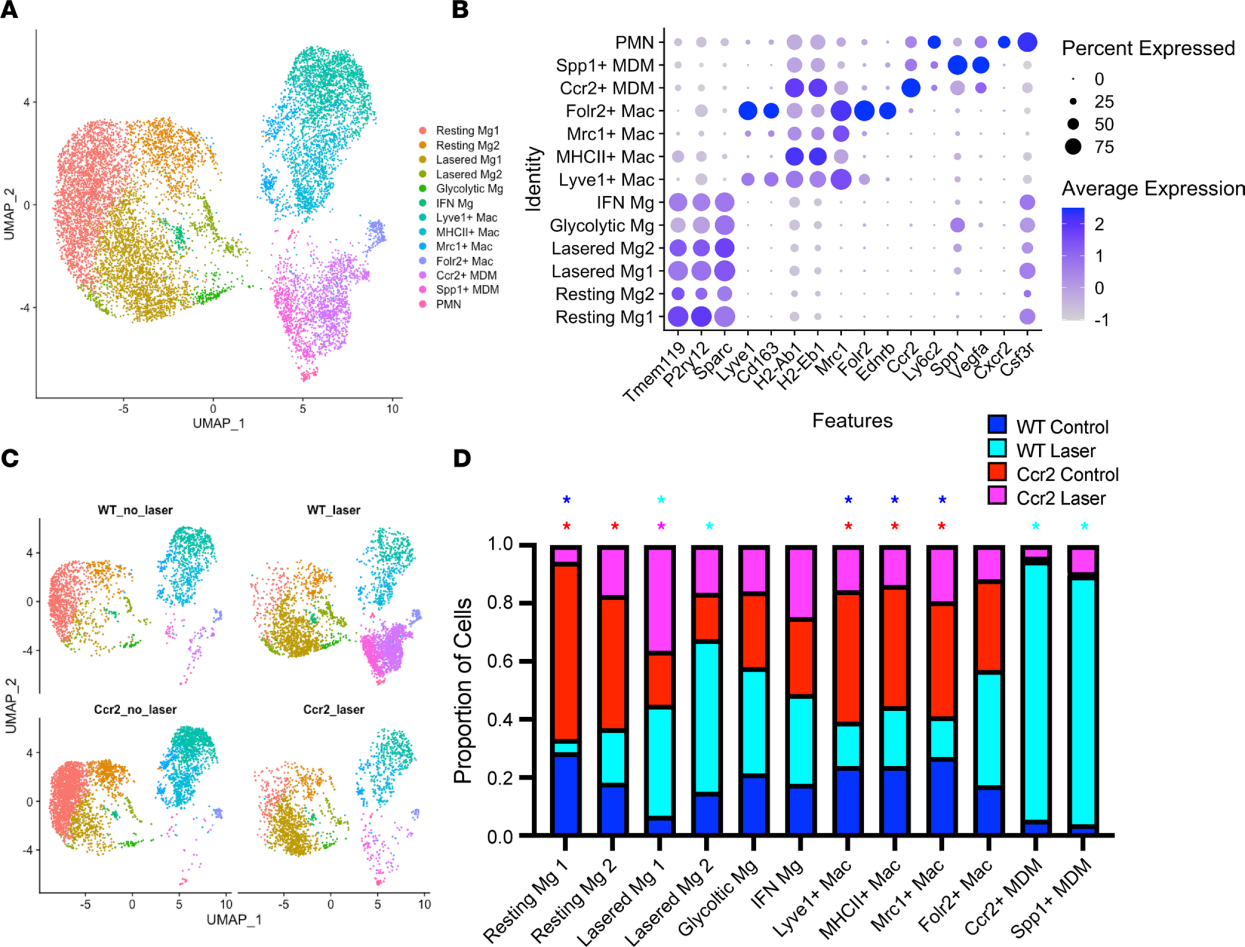
Single-cell identification of ocular macrophages. (**A**) UMAP dimension plot. (**B**) Dot plot of marker genes for each cell type. (**C**) UMAP dimension plot split by experimental group. (**D**) Proportion of cells derived from each experimental group. *P < 0.001 by hypergeometric enrichment test. Color of asterisk identifies which groups were enriched.

We identified 12 Mac cell clusters including 6 Mg clusters, 4 Mac populations, 2 MDM subsets, and 1 minor group of doublets including both PMNs (*Cxcr2*^+^*Csf3r*^+^) and Macs (Figure 3, A and B). The 6 Mg clusters all specifically expressed *Tmem119*, *P2ry12*, and *Sparc* Mg markers (Figure 3B). Among the 4 Mac clusters, we found Lyve1^+^ Mac (defined as *Lyve1*^+^*Cd163*^+^), MHCII^+^ Mac (*H2-Ab1*^+^*H2-Eb1*^+^), Mrc1^+^ Mac (*Mrc1^+^*), and Folr2^+^ Mac (*Lyve1*^+^*Cd163*^+^*Folr2*^+^*Ednrb*^+^) populations (Figure 3B). The MDM populations included MHCII^+^*Ccr2*^+^ and *Vegfa*^+^*Spp1*^+^ MDMs, which we named Ccr2^+^ and Spp1^+^ MDM, respectively. The proportional contribution from the 4 experimental groups to each cluster is shown in Figure 3D, and the proportion of cells that each cluster contributes to the 4 experimental groups is shown in Supplemental Table 3. We used a hypergeometric distribution analysis to determine which genotype and treatment specifically contributed to each cluster. This analysis identified 3 Mac subtypes that were enriched from WT and *Ccr2^–/–^* unlasered groups (Figure 3, C and D), including Lyve1^+^ Mac, MHCII^+^ Mac, and Mrc1^+^ Mac (Figure 3D). These subtypes likely included iris and ciliary body Macs (Lyve1^+^ Mac) and choroidal Macs (MHCII^+^ Mac), based upon prior publications (15, 26–28). Folr2^+^ Mac was not enriched from any group and could include perivascular Macs and/or hyalocytes based upon its expression of *Lyve1* and *Mrc1*/CD206 (29). Hypergeometric distribution analysis also delineated 2 MDM subsets that were specifically derived from the WT + laser group: Ccr2^+^ MDM and Spp1^+^ MDM (Figure 3, C and D). Ccr2^+^ MDM cells were MHCII^+^*Ccr2*^+^, confirming that our study design identified C-MDMs (Figure 3B). The Spp1^+^ MDM cluster was increased 13.2-fold by laser in WT mice, and this effect was ablated in *Ccr2^–/–^* mice (Figure 3, C and D). Additionally, Spp1^+^ MDMs expressed the most *Vegfa*, suggesting that this cluster could be our sought-after disease-associated Mac subtype (Figure 3B). In support of this hypothesis, Spp1^+^ Macs have recently been linked to both experimental CNV and nAMD in patients (30, 31).

**Figure 4:**
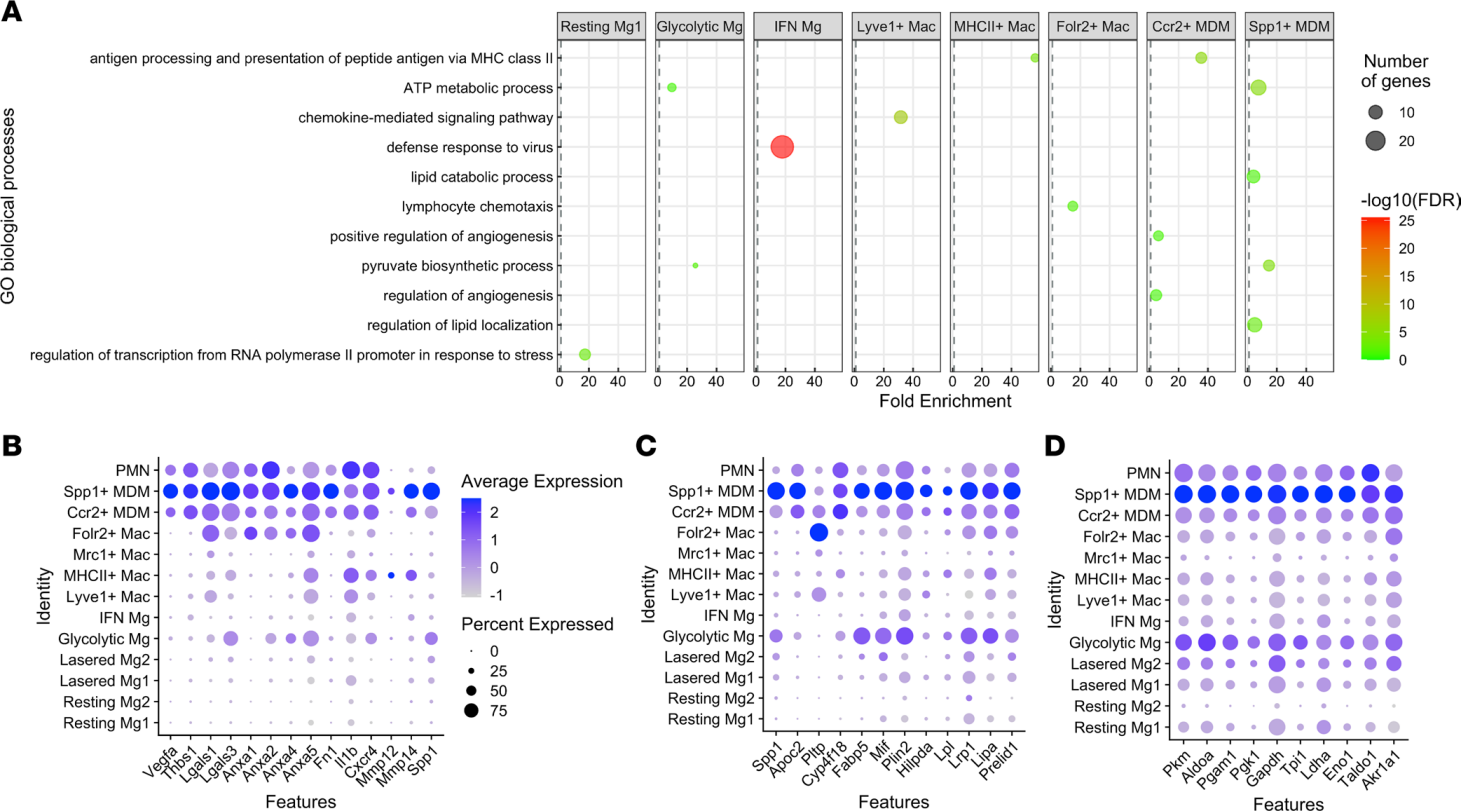
Gene ontology (GO) enrichment analysis of ocular macrophages. (**A**) Graph of GO enrichment from each macrophage cluster visualizing GO term on the *y* axis, fold enrichment on the *x* axis, FDR *q* value in color, and number of genes as the size of the circle. (**B**–**D**) Dot plot graphs of genes that were differentially expressed in the positive regulation of angiogenesis (**B**), lipid catabolism and localization (**C**), and glycolysis (**D**) GO terms.

To gain insight into the function of each Mac subset, we performed differential expression analysis comparing each Mac cluster with all other clusters (fold change > 2 and *P* < 0.001). Next, we used GOrilla gene ontology (GO) analysis (32) to infer the function of each MDM subset (FDR *q* <0.05). We found that Ccr2^+^ MDMs were enriched for positive regulation of angiogenesis (6.3-fold, *q* < 0.05) and regulation of angiogenesis (4.76-fold, *q* < 0.05) GO terms (Figure 4A). Genes that were upregulated in Ccr2^+^ MDMs that contribute to angiogenesis included *Vegfa* (2.2-fold), *Lgals3* (Galectin-3, 3.6-fold), *Anxa1* (Annexin-A1, 2.5-fold), *Fn1* (Fibronectin, 3.5 fold), *Il1b* (IL-1β, 2.5-fold), and *Cxcr4* (2.3-fold) (Figure 4B). Although Spp1^+^ MDMs were not enriched for angiogenesis GO terms, each of the DE genes that stimulated angiogenesis in Ccr2^+^ MDMs were further increased in Spp1^+^ MDMs (Figure 4B). For example, *Vegfa* (3.7-fold), *Lgals3* (11.8-fold), *Anxa1* (3.2-fold), and *Fn1* (10.1-fold) showed larger fold changes in Spp1^+^ MDMs compared with Ccr2^+^ MDMs. Furthermore, additional proangiogenic DE genes — including *Lgals1*, *Anxa2/4/5*, *Mmp12*, and *Mmp14* — were upregulated in Spp1^+^ MDMs (Figure 4B). Angiogenesis GO terms were not statistically significant in Spp1^+^ MDMs because there were more total differentially expressed genes compared with Ccr2^+^ MDMs (130 versus 58), preventing the angiogenesis-related genes from being statistically enriched due to more total genes. Therefore, Spp1^+^ MDMs express a transcriptome consistent with angiogenesis development through a multimodal pathway including VEGF signaling, endothelial sprouting via annexins (33), galectin receptor–driven increases in TGF-β and VEGF signaling (34), cytokine signaling (35, 36), and *Mmp12*-stimulated fibrosis (37).

In support of the hypothesis that Spp1^+^ MDMs are proangiogenic, Spp1^+^ MDMs were also enriched for regulation of lipid transport (4.5-fold, *q* < 0.05), regulation of lipid localization (4.9-fold, *q* < 0.01), and lipid catabolic process (4.1-fold, *q* < 0.05) (Figure 4, A and C). Cholesterol-laden Macs have been previously implicated as proangiogenic Macs in experimental CNV (38, 39). Additionally, Spp1^+^ MDMs were enriched for pyruvate biosynthetic process and glycolytic process (14.6-fold, *q* < 0.001 for both; Figure 4A). Nearly every gene involved in glycolysis was upregulated in Spp1^+^ MDMs compared with all other Macs (Figure 4D). Importantly, glycolytic Macs are linked with both retinal and choroidal angiogenesis (40, 41). Therefore, Spp1^+^ MDMs are derived from CMos, are linked to CNV and nAMD in mice and humans, express a multimodal proangiogenic transcriptome, are cholesterol laden, and are proglycolytic; these characteristics link Spp1^+^ MDMs to disease-associated proangiogenic Macs.

**Figure 5:**
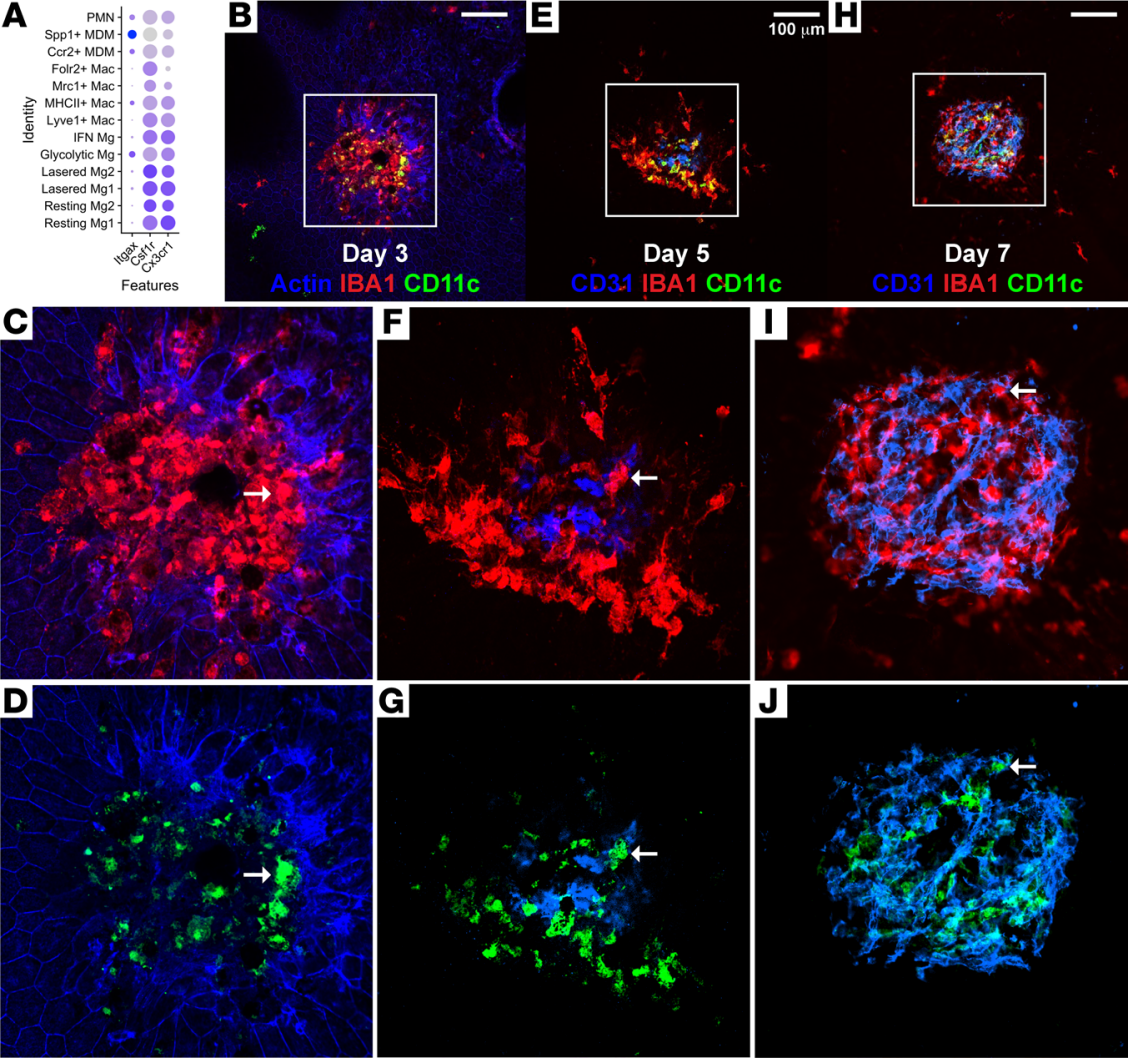
Spp1^+^ MDMs express CD11c, and CD11c^+^ macrophages are present prior to and during laser CNV growth. (**A**) Dot plot graph of *Itgax*/CD11c expression in each macrophages cluster. (**B**–**J**) IF imaging of laser CNV lesions from choroidal whole mounts using *CD11c^Cre–eGFP^* mice. (**B**–**D**) On Day 3, GFP^+^IBA1^+^ and GFP^–^IBA1^+^ macrophages were present at laser injury sites (lack of hexagonal Actin^+^ RPE cells). (**E**–**G**) On Day 5, GFP^+^IBA1^+^ macrophages were near early CD31^+^ neovascular lesions. (**H**–**J**) On Day 7, GFP^+^IBA1^+^ macrophages were present in mature CD31^+^ neovascularizations. White boxes indicate magnified areas in **C**, **D**, **F**, **G**, **I**, and **J** insets. White arrows indicate GFP^+^IBA1^+^ colocalizing cells. Scale bars: 100 μm (**B**, **E**, and **H**).

To target proangiogenic Spp1^+^ MDMs, we queried our scRNA-Seq data to identify unique cell surface receptors for Spp1^+^ MDMs. Spp1 is a secreted protein and, thus, would be a poor cellular marker. *Csf1r* and *Cx3cr1* are both universal monocyte and Mac markers found in monocytes, Mg, and Mac subtypes (Figure 5A). *Itgax*/CD11c was expressed in Spp1^+^ MDMs at greater levels than any other Mac cluster (Figure 5A). In support, we previously showed that CD11C^+^ Macs are derived from patients with nAMD and express a proangiogenic transcriptome in scRNA-Seq data from human choroid (15). Because our scRNA-Seq data are from whole mouse eyes, it is important to determine if CD11c^+^ Macs are present in mouse CNV lesions. Ten- to 12 week-old CD11c^Cre-GFP^ mice underwent laser injury, followed by IF imaging of choroidal whole mounts at Days 3 (the peak of Mac infiltration), Day 5 (early CNV development), and Day 7 (peak CNV size). We found that GFP^+^IBA1^+^ Macs were present in CNV lesions at all 3 time points (Figure 5, B–J).

**Figure 6:**
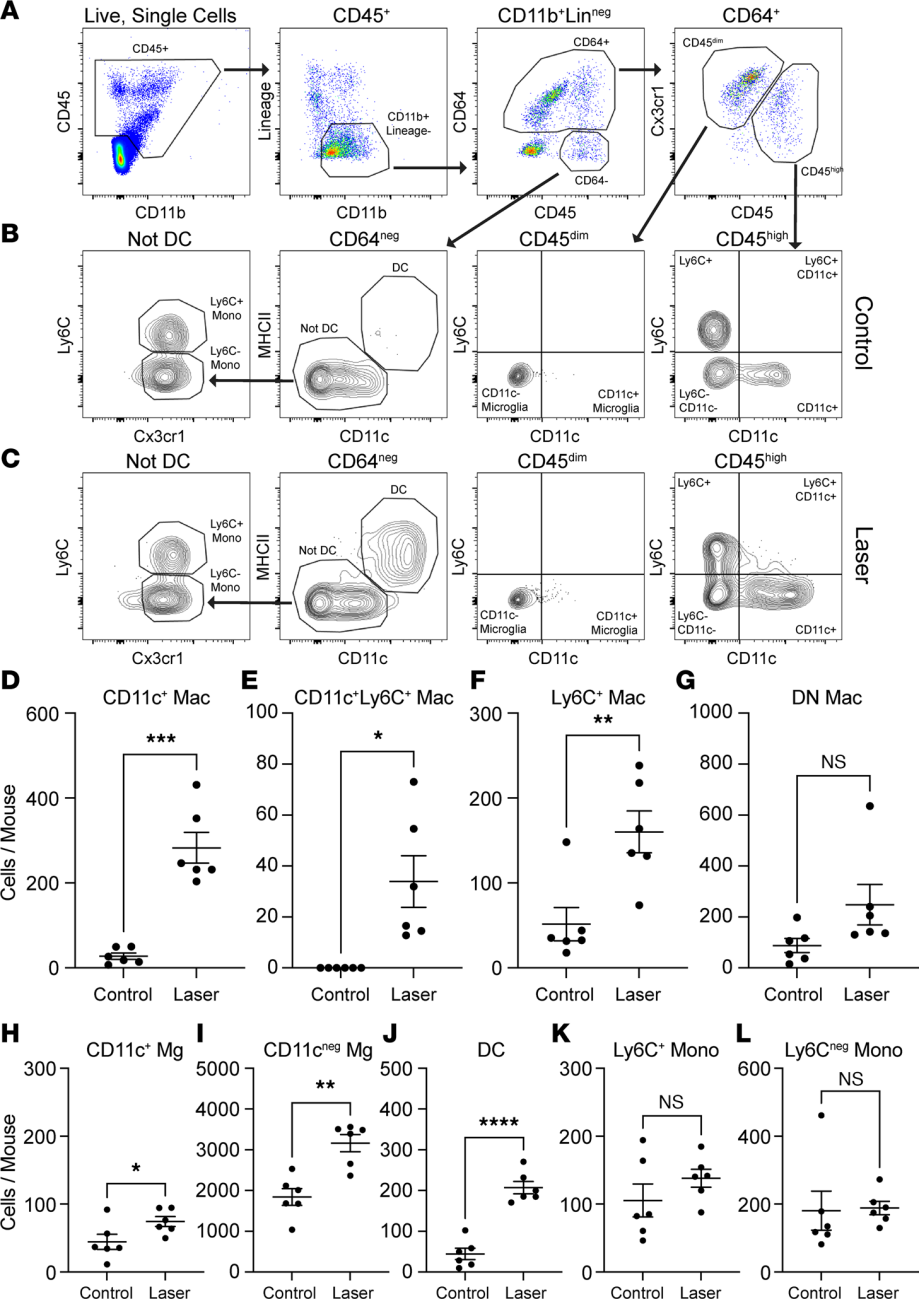
CD11c^+^ macrophages are significantly increased by laser injury. (**A**) Flow cytometry gating strategy. Left panel: CD45^+^ cells were identified from Live, Singlet cells identically to Figure 1B. Middle left panel: Lineage (CD4, CD8 [T cells], B220 [B cells], Ly6G [neutrophils], NK1.1 [NK cells], SiglecF [eosinophils]) versus CD11b plot to gate forward CD11b^+^Lin^–^ mononuclear phagocytes. Middle right panel: CD64^+^ macrophages were discriminated from CD64^–^ monocytes and DCs. Right panel: Cx3cr1 versus CD45 plot to delineate Cx3cr1^hi^CD45^dim^ microglia from CD45^hi^ infiltrating macrophages. (**B** and **C**) Flow cytometry gating strategy for the identification of macrophage and microglia subtypes, DCs, and monocytes from control (**B**) and laser-treated (**C**) choroid and retina. Right panel: CD45^hi^ infiltrating macrophages were separated into 4 groups: CD11c^+^, CD11c^+^Ly6C^+^, Ly6C^+^, and double-negative (DN) macrophages. Middle right panel: CD45dim microglia were divided into 2 groups: CD11c^+^ and CD11c^–^ microglia. Middle left panel: DCs were identified as CD64^–^MHCII^+^CD11c^+^ cells. Left panel: Ly6C versus Cx3cr1 plot from Not DC cells to delineate Ly6C^+^Cx3cr1^+^ classical monocytes and Ly6C^–^Cx3cr1^+^ nonclassical monocytes. (**D**–**F** and **H**–**J**) Quantitative analysis of mononuclear phagocyte cell numbers after laser treatment. CD11c^+^ macrophages (**D**, Welch’s *t* test), CD11c^+^Ly6C^+^ macrophages (**E**, Welch’s *t* test), Ly6C^+^ macrophages (**F**, unpaired *t* test), CD11c^+^ microglia (**H**, unpaired *t* test), CD11c^–^ microglia (**I**, unpaired *t* test), and DCs (**J**, unpaired *t* test) were significantly increased in the choroid and retina after laser treatment (*n* = 6 per group for all). (**G**, **K**, and **L**) DN macrophage (**G**, Welch’s *t* test), classical monocyte (**K**, unpaired *t* test), and nonclassical monocytes (**L**, Welch’s *t* test) numbers were not significantly increased after laser treatment. *P < 0.05, **P < 0.01, ***P < 0.001, ****P < 0.0001.

Next, we performed multiparameter flow cytometry to quantitate CD11c^+^ Macs during laser-induced CNV in choroid complex (choroid/ retinal pigment epithelium [RPE]/ sclera) and retina. Choroid complex and retina were chosen because the CNV lesion exists between the retina and choroid. Infiltrating immune cells are found in both the retina and choroid complex after dissection in our prior studies. WT C57BL/6J mice underwent laser injury, and eyes were harvested on Day 3. Eyes were enucleated and dissected to isolate retina and choroid/RPE/sclera complex for multiparameter flow cytometry analysis. Live, singlet CD45^+^ cells were identified (Figure 6A, left). Mononuclear phagocytes (CD11b^+^Lin^–^) were delineated by gating forward CD11b^+^ cells but excluding Lin^+^ (Lin: T cells, B cells, NK cells, eosinophils, and neutrophils) cells. CD64 expression was used to isolate Macs from monocytes and DCs (Figure 6A, middle right). CD45 and Cx3cr1 expression differentiated CD64^+^CD45^dim^Cx3cr1^hi^ Mg from infiltrating CD64^+^CD45^hi^ Macs (Figure 6A, right). We discriminated 4 infiltrating Mac populations: CD11c^+^, CD11c^+^Ly6C^+^, Ly6C^+^, and double-negative (DN) Macs (Figure 6, B and C, right). After laser injury, CD11c^+^ and Ly6C^+^ Macs were elevated by 10.2-fold (*P* < 0.001, Figure 6D) and 3.1-fold (*P* < 0.01, Figure 6F), respectively. Additionally, CD11c^+^Ly6C^+^ Mac numbers significantly increased from 0 to 33.9 ± 10.1 cells in lasered mice (*P* < 0.0001; Figure 6E). Alternatively, only a trend toward greater CD11c^–^Ly6C^–^ Macs was detected (Figure 6G). Mg did not express Ly6C, as expected, and were parsed into CD11c^–^ and CD11c^+^ Mg (Figure 6, B and C, middle right). CD11c^+^ (*P* < 0.05; Figure 6H) and CD11c^–^ (*P* < 0.01; Figure 6I) Mg numbers each grew by 1.7-fold. From CD64^–^ cells, DCs were identified as MHCII^+^CD11c^+^ (Figure 6, B and C, middle left), and were significantly increased 4.6-fold by laser injury (*P* < 0.0001; Figure 6J). Finally, from non-DCs, we delineated Ly6C^+^Cx3cr1^+^ and Ly6C^–^Cx3cr1^+^ monocytes (Figure 6, B and C, left). Neither monocyte population was significantly influenced by laser injury (Figure 6, K and L). In summary, our flow cytometry analysis confirmed both our scRNA-Seq and IF data indicating that CD11c^+^ Macs are significantly increased in the retina and choroid/RPE/sclera complex during the laser-induced CNV model.

**Figure 7:**
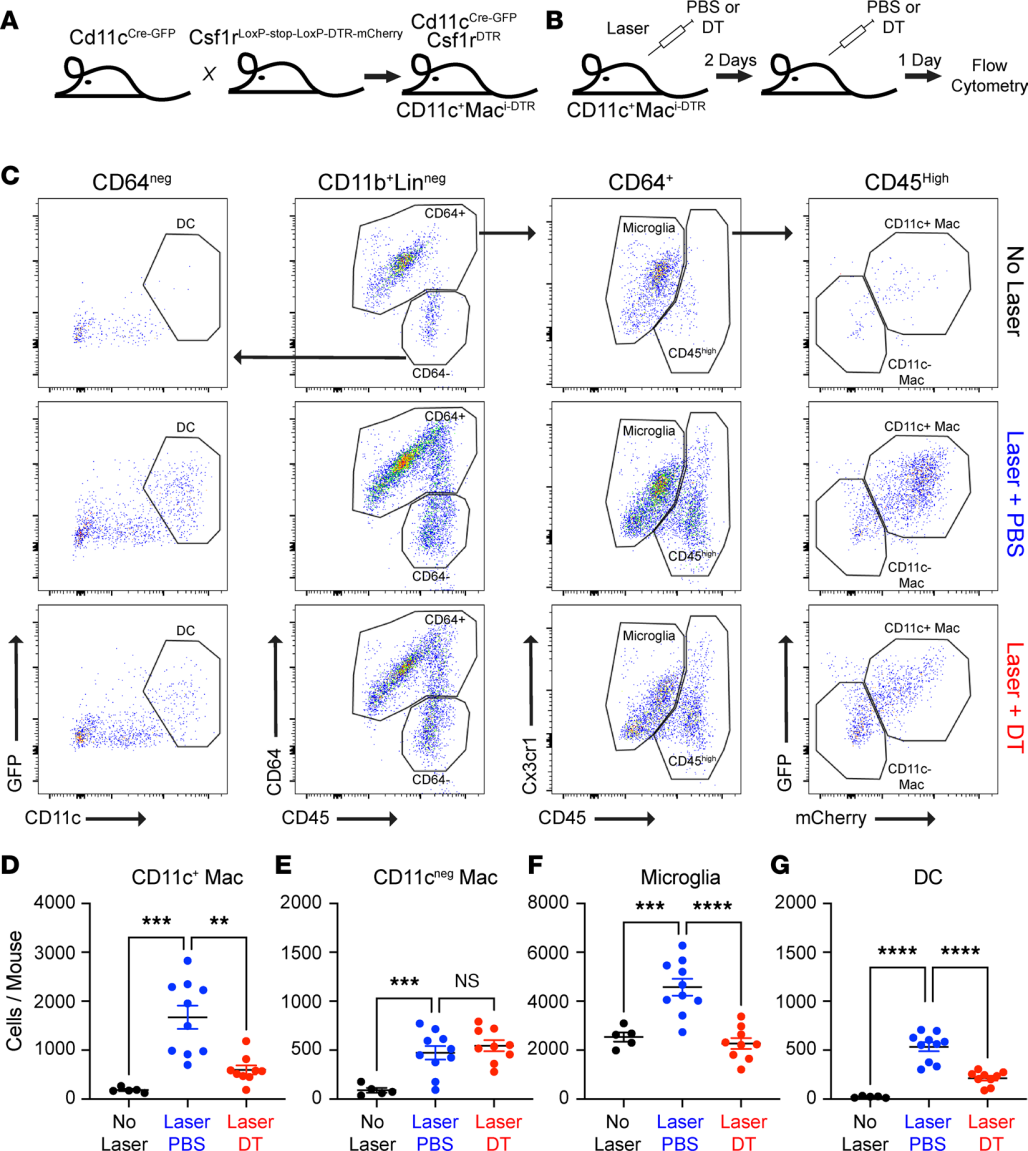
CD11c^+^Mac^iDTR^ mice deplete CD11c^+^ macrophages. (**A**) Breeding strategy to generate CD11c^+^Mac^iDTR^ mice. (**B**) Schematic of experimental design. (**C**) Flow cytometry gating strategy. Top: control no laser. Middle: Laser + PBS. Bottom: Laser + DT. Middle left panel: CD11b^+^Lin^–^ cells were identified identically to Figure 6A. CD64 was used to differentiate macrophages from nonmacrophage populations. Middle right panel: Cx3cr1 versus CD45 plot to discriminate CD45^dim^ microglia from CD45^hi^ macrophages. Right panel: GFP versus mCherry plot to separate GFP^+^mCherry^+^CD11c^+^ macrophages from GFP^–^mCherry^–^CD11c^–^ macrophages. Left panel: GFP versus CD11c plot to identify GFP^+^CD11c^+^ DCs. (**D**–**G**) Quantitative analysis of mononuclear phagocyte groups. (**D**) CD11c^+^ macrophages were increased 9-fold in the laser + PBS group and depleted by 65% by DT. (**E**) CD11c^–^ macrophages were increased by laser and unaffected by DT. (**F** and **G**) Microglia (**F**) and DCs (**G**) were increased by laser and also depleted by DT treatment. *n* = 5–10 per group for all. Brown-Forsythe and Welch’s ANOVA followed by Dunnett’s T3 multiple comparisons test. **P < 0.01, ***P < 0.001, ****P < 0.0001.

**Figure 8:**
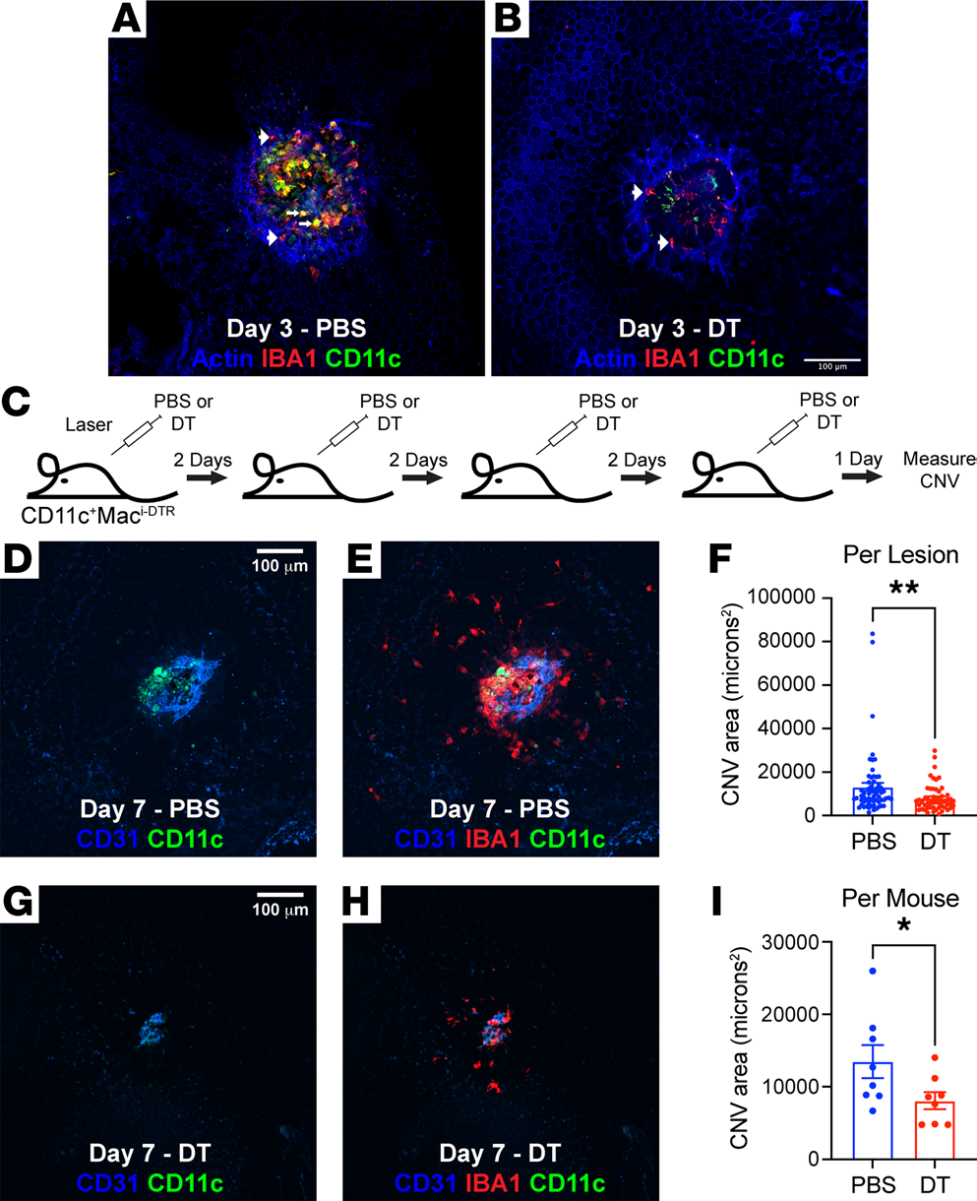
CD11c^+^Mac^iDTR^ mice have reduced CNV area. (**A** and **B**) IF imaging of choroidal whole mounts on Day 3 after laser injury from CD11c^+^Mac^iDTR^ mice. (**A**) In PBS-treated mice, both GFP^–^IBA1^+^ (arrowheads) and GFP^+^IBA1^+^ (white arrows) macrophages were present at laser injury sites. (**B**) In DT-treated mice, GFP^–^IBA1^+^ macrophages (arrowheads) and GFP^+^IBA1^–^ DCs were present, but GFP^+^IBA1^+^ macrophages were depleted. (**C**) Schematic of experimental design for laser CNV analysis. (**D** and **E**) Representative IF image on Day 7 from a PBS-treated mouse. GFP^+^IBA1^+^ macrophages were found on Day 7 in the CD31^+^ CNV lesion. (**G** and **H**) Representative IF image on Day 7 from a DT-treated mouse. GFP^+^IBA1^+^ macrophages were depleted by DT and CD31^+^ CNV lesion area was decreased. (**F** and **I**) Quantitative analysis of CNV area between PBS- and DT-treated mice. DT treatment reduced CNV area by 40% in both per lesion (*n* = 54–62 per group, **P < 0.01, Mann Whitney *U* test) and per mouse (*n* = 8 per group, *P < 0.05, Mann Whitney *U* test) analysis. Scale bars: 100 μm.

We have demonstrated that CD11c^+^ Macs are increased by laser, recruited specifically to CNV sites, and express a proangiogenic, proglycolytic, and prolipid catabolic transcriptome. Therefore, we tested the hypothesis that CD11c^+^ Macs are pathogenic during the laser-induced CNV model by creating a genetic model to ablate CD11c^+^ Macs. We bred *CD11c^Cre-GFP^* and *Csf1r^LoxP-stop-LoxP-DTR-mCherry^* mice to generate *CD11c^+^Mac^iDTR^* mice. In *CD11c^+^Mac^iDTR^* mice, *Itgax*/CD11c-expressing cells will also express Cre recombinase, which will excise the stop codon and allow DT receptor (DTR) and mCherry expression in *Csf1r*-expressing cells (Figure 7A). Female 10- to 12-week-old *CD11c^+^Mac^iDTR^* mice underwent laser injury followed by PBS or DT treatment on Day 0 and Day 2. Eyes were harvested on Day 3 for multi-parameter flow cytometry of retina and choroid/RPE/sclera complex (Figure 7B). Single, live, CD45^+^CD11b^+^Lin^–^ cells were gated forward, identically to Figure 6, A and B. CD64 expression was used to separate DCs/monocytes from Macs (Figure 7C, middle left). DCs were delineated as CD64^–^GFP^+^CD11c^+^ (Figure 7C, left). Mg were identified as CD64^+^CD45^dim^, and infiltrating Macs were identified as CD64^+^CD45^hi^ (Figure 7C, middle right). Infiltrating Macs were separated into GFP^+^mCherry^+^CD11c^+^ Macs and GFP^–^mCherry^–^CD11c^–^ Macs (Figure 7C, right). We found CD11c^+^ Macs were increased 9.0-fold by laser compared with control eyes (*P* < 0.001) and were reduced 65% by DT treatment (*P* < 0.01; Figure 7D). Alternatively, CD11c^–^ Macs were elevated 5.2-fold (*P* < 0.001) by laser injury compared with unlasered eyes and unchanged after DT treatment (Figure 7E). Mg numbers grew by 1.8-fold in the laser group (*P* < 0.001) and were reduced back to baseline after DT treatment (*P* < 0.0001; Figure 7F). Finally, DCs increased by 23.6-fold after laser treatment (*P* < 0.0001) and were decreased 60% by DT (*P* < 0.0001, Figure 7G). Using IF imaging, we confirmed that CD11c^+^ Macs are present in CNV lesions on Day 3 (white arrows) and are depleted by DT treatment (Figure 8, A and B). Additionally, IBA1^+^CD11c^–^ Macs (white arrowheads) were still present at CNV sites in PBS- and DT-treated mice (Figure 8, A and B). These data demonstrate that *CD11c^+^Mac^iDTR^* mice are a suitable model to ablate CD11c^+^-infiltrating Macs without affecting CD11c^–^ Macs.

Next, we tested if DT treatment decreased laser-induced CNV area in *CD11c^+^Mac^iDTR^* mice. Female 10- to 12-week-old *CD11c^+^Mac^iDTR^* mice underwent laser injury followed by PBS or DT treatment every 2 days. On Day 7, choroidal whole mounts were harvested to measure CNV area by CD31 staining (Figure 8C). On Day 7, CD11c^+^IBA1^+^ Macs were detected at CNV sites in PBS-treated (Figure 8, D and E) and absent from DT-treated (Figure 8, G and H) mice. Average CNV lesion area was reduced by 40% with DT treatment (*P* < 0.01, *n* = 54–62 lesions; Figure 8F). Average CNV lesion area per animal was also decreased by 40% (*P* < 0.05, *n* = 8 mice; Figure 8I). These data suggest that CD11c^+^ Macs are necessary for laser-induced CNV pathogenesis.

## Discussion

Using scRNA-Seq analysis of whole mouse eyes undergoing laser-induced CNV, we uncovered 12 ocular Mac populations from WT and *Ccr2^–/–^* eyes. These 12 Mac groups separated into 6 Mg subsets: 4 Mac clusters, including potential MHCII^+^ choroidal Macs and Lyve1^+^ ciliary body Macs, and 2 MDM subtypes that were enriched from lasered WT eyes (Figure 3). Differential expression and GO analysis found that Spp1^+^ MDMs expressed high levels of genes that positively regulate angiogenesis, increase glycolysis, and catabolize and store lipids (Figure 4). To validate these findings, we found that Spp1^+^ Macs express high levels of *Itgax*/CD11c. We performed IF and multiparameter flow cytometry to demonstrate that CD11c^+^ Macs are increased 10-fold in the retina and choroid/RPE/sclera complex after laser injury and are present at CNV sites from Days 3–7 (Figures 5 and 6). Finally, we generated CD11c^+^ Mac depleting mice and demonstrated that DT treatment reduced CD11c^+^ Macs by 65% and reduced laser-induced CNV area by 40% (Figures 7 and 8). Thus, CD11c^+^ Macs are necessary for CNV. It is likely that occurs by stimulating a multimodal proangiogenic gene expression program, including increased *Vegfa* expression directly, galectin receptor-driven increased TGF-β and VEGF signaling (34), endothelial sprouting via annexins (33), cytokine (via *Il1b* and *Cxcr4*) signaling (35, 36), and *Mmp12* stimulated fibrosis (37).

scRNA-Seq of enriched endothelial cells from the laser-induced CNV model identifies both common proangiogenic pathways and interactions between CD45^+^ cells and CNV-specific endothelial cells. Endothelial cells differentiate during CNV in pseudotime trajectory as follows: activated postcapillary venules, transitioning endothelial cells, both proliferating and immature cells, tip cells, and stalk cells (42). Activated postcapillary venules specifically express *Cxcl1* and *Csf3* (42) — the ligands for CXCR2 and CSF3R, which were specifically expressed by neutrophils in our data set (Figure 1). Neutrophils can promote CNV (43), and their early recruitment by activated postcapillary venules may stimulate further Mac infiltration. Transitioning endothelial cells specifically express *Vcam1* and *Icam1* (42), which promote leukocyte adhesion via CD11b (a receptor expressed on all Macs). Additionally, ICAM1 or CD11b ablation inhibits CNV (44). Proliferating, immature, and tip endothelial cells were enriched for glycolytic metabolism (42), a common metabolic pathway with CD11c^+^ Macs (Figure 4). Tip cells also specifically expressed *Lgals1* in common with CD11c^+^ Macs and *Cd63*, which promotes VEGF signaling (*Vegfa* is expressed by CD11c^+^ Macs; Figure 4), through its role on VEGFR2 internalization and activation (45). Finally, stalk cells specifically express *Cxcl12* and *Kdr*/VEGFR2 (42); the importance of VEGF-VEGFR2 interactions are well known. CXCR4 (receptor for CXCL12) is specifically expressed by CD11c^+^ Macs (Figure 4) and its inhibition reduces CNV (46). These interactions with endothelial cells demonstrate that CD11c^+^ Macs may crosstalk with endothelial cells at multiple stages of CNV differentiation.

Our study is the second scRNA-Seq analysis to our knowledge of immune cells from the laser-induced CNV model to date (47). However, a significant difference between the 2 studies was that Wieghofer et al*.* processed retina while we harvested whole eyes for FACS and scRNA-Seq. Wieghofer et al*.* identified predominantly Mg by scRNA-Seq; this is not surprising, considering that the tissue harvested only consisted of retina. However, as evidenced by Figures 5 and 8, retina-lacking choroidal whole mounts contain many Macs at the laser site, which were not sequenced by Wieghofer et al. and were captured by our methodology. Additionally, we sequenced 34,215 cells compared with 1,536 cells in Wieghofer et al*.* Therefore, our identification of a proangiogenic MDM population is not surprising, considering our greater number of cells, ability to detect rarer cell types, and inclusion of choroidal tissue. Additionally, our finding that CD11c^+^Spp1^+^ MDMs increase CNV area is in agreement with the multiple laboratories that have shown that *Ccr2^–/–^* mice show decreased CNV area by ~50% (14–16). This is similar to our 40% effect size, considering that we only ablated 65% of CD11c^+^ Macs that were increased by laser treatment.

Cholesterol-laden, proglycolytic, CD11c^+^, and/or Spp1^+^ Macs have been previously linked to laser-induced CNV in mice and/or nAMD in patients. In patients with the 10q26 risk allele for AMD, high HTRA1 serine peptidase expression cleaves thrombospondin 1, preventing CD47 activation and increasing *SPP1* expression in Macs (30). Additionally, *Spp1^–/–^* mice show reduced Mac accumulation and decreased CNV area (30). Furthermore, SPP1^+^ Macs are detectable in human CNV membranes (31). These data link Spp1^+^ Macs to CNV and nAMD. We previously analyzed scRNA-Seq data from human choroidal tissue of healthy patients and patients with nAMD (28). We found that CD11C^+^ Macs were enriched from patients with nAMD and expressed a proangiogenic transcriptome, which included several overlapping genes to Spp1^+^ MDMs, including *VEGFA*, *CXCR4*, and *IL1* (15). In addition to *Spp1* and CD11c, Macs with increased glycolytic flux are detectable in retinal neovascularization from the oxygen-induced retinopathy model and during laser-induced CNV. Furthermore, inhibition of glycolysis in Mac-specific *Pfkfb3^–/–^* mice reduced angiogenesis during oxygen-induced retinopathy and CNV (40, 41). Finally, cholesterol-laden Macs, which are increased in aged mice, are associated with anti-VEGF resistance and larger CNV lesions (38, 39). Taken together, there is strong consensus in the literature that supports our finding that CD11c^+^Spp1^+^ glycolytic, lipid-laden Macs stimulate angiogenesis during CNV.

To further validate this finding, we generated CD11c^+^ Mac–depleting (*CD11c^+^Mac^iDTR^*) mice by breeding *CD11c^Cre-GFP^* and *Csf1r^LoxP-stop-LoxP-DTR-mCherry^* mice. In *CD11c^+^Mac^iDTR^* mice, CD11c^+^ and CD11c^–^ Macs were increased by laser, but only CD11c^+^ Macs were depleted by DT treatment. However, DT treatment also reduced Mg and DCs in *CD11c^+^Mac^iDTR^* mice. Therefore, it is possible that DCs or Mg could contribute to our 40% reduction in CNV size in DT-treated *CD11c^+^Mac^iDTR^* mice. We previously showed that *Flt3^–/–^* and *Flt3-ligand^–/–^* mice have no change in CNV area (48), suggesting that DCs are not significant contributors. However, we cannot rule out the possibility that the 1.8-fold increase in Mg after laser, which was ablated by DT treatment, reduces CNV area. Since Mg can express both *Itgax*/CD11c and *Csf1r*, some Mg depletion was expected. In fact, CD11c is a marker for disease-associated Mg (49), which were increased by laser injury and would be the likely Mg subtype involved in CNV pathogenesis. While the role of Mg in laser-induced CNV is a subject of debate, it is possible that subretinal disease-associated Mg could protect photoreceptors from degeneration or could pathologically contribute to angiogenesis. Mg-depleting experiments using specific promoters like *Tmem119^CreER^* or *P2ry12^CreER^* have not been performed to definitively determine the role of Mg in the laser-induced CNV model. Future experiments are warranted to clarify the role of Mg and, specifically, disease-associated Mg in CNV.

This study has several important limitations. We acknowledge that it would have been ideal to perform our scRNA-Seq and multiparameter flow cytometry studies on individual tissues like retina and choroid complex independently or to specifically dissect the retinal and choroidal regions impacted by laser. These design improvements are opportunities to gain more detailed insights into the role of the innate immune system during laser-induced CNV. However, we performed scRNA-Seq on whole eyes from 9–15 mice per group to increase our CD45^+^ cell yield to sequence 10,000 cells per group. Parsing the eyes into individual tissues, although ideal, would have significantly reduced our cellular yield, increased our number of necessary animals, and increased the cost of sequencing. We confirmed the presence of proangiogenic CD11c^+^ Macs at laser CNV lesions by IF imaging of the CNV lesions and by multiparameter flow cytometry. We performed our flow cytometry on the retina and choroid complex together because the CNV lesion exists between the retina and choroid, causing dissection to split the infiltrating immune cells between tissues. Parsing the posterior eye cup into individual tissues, however, could have gained more detailed insights. It is also noteworthy that cDC2 cells were recently shown to express CD64/*Fcgr1* (50), in agreement with our findings in Figure 2, albeit at lower levels than Macs. Therefore, it is possible that our gating strategy for CD11c^+^ Macs could have included cDC2s. Finally, the laser-induced CNV model has a larger and more acute inflammatory component compared with nAMD.

In summary, we have identified Spp1^+^CD11c^+^ MDMs that express a transcriptome-promoting glycolysis, lipid catabolism, and angiogenesis by multiple VEGF-dependent and VEGF-independent pathways using scRNA-Seq from mouse eyes. We confirmed that CD11c^+^ Macs are increased after laser in the retina and choroid/RPE/sclera complex by flow cytometry and are present at CNV sites by IF. Finally, we created a genetic model to deplete CD11c^+^ macrophages, and this model decreased laser-induced CNV area by 40%. These data demonstrate that CD11c^+^ Macs stimulate angiogenesis and are necessary for laser-induced CNV. Novel therapeutics to target Mac subtypes, potentially with a bispecific antibody, are an unmet need for anti-VEGF resistant patients.

## Methods

### Animals.

All animal studies were performed on 10- to 12-week-old female mice. Original breeders for WT animals (C57BL/6J; no. 000664), *Ccr2*^–/–^ (no. 004999), *Csf1r^LoxP-stop-LoxP-DTR-mCherry^* (no. 024046), and *CD11c^Cre–eGFP^* (no. 007567) were purchased from The Jackson Laboratory and bred in-house. *Csf1r^LoxP-stop-LoxP-DTR-mCherry^* were bred with *CD11c^Cre–eGFP^ mice* to generate *CD11c^+^Mac^iDTR^* mice. Animals were housed in a barrier facility in the Northwestern University Center for Comparative Medicine under a 12-hour light/dark cycle with unlimited access to food and water. Genotypes of all mice, including the absence of the *Rd8* mutation, were confirmed by services provided by Transnetyx Inc.

### Laser-induced CNV model.

CNV was induced as previously described (51). Briefly, mice were anesthetized and eyes were dilated and lasered with a slit lamp (Zeiss) mounted ophthalmic laser (IRIDEX). Each eye was treated with either 4 (CNV area quantification) or 8 (scRNA-Seq and flow cytometry analysis) focal laser burns (75 μm, 110 mW, 100 msec). Eyes with major bleeding at the time of laser were excluded. In Mac-depleting *CD11c^+^Mac^iDTR^* mice, we i.p. injected 4 ng DT/g (MilliporeSigma, D0564, dissolved in PBS) at the time of laser injury and for every 2 days thereafter.

### Sample preparation for scRNA-Seq.

Single-cell suspensions of whole digested eyes were obtained as previously described (22). Prior to any staining, cell suspensions were subjected to a CD45 enrichment using the protocol provided by the manufacturer (Miltenyi Biotec, 130-052-301). Enriched, live cells were stained for CD45 and CD11b (Table 1) and sorted on a BD FACSAria cell sorter (BD Biosciences) at the Northwestern University RHLCCC Flow Cytometry Facility. Sorted populations were collected in MACS buffer (Miltenyi Biotec, 130-091-221). Cells were centrifuged at 350*g* for 10 minutes at 4°C and resuspended in RPMI (MilliporeSigma, R8758). scRNA-Seq libraries were prepared as previously described (52) using the Single Cell 3′ v3 Reagent Kit. Gel Bead in emulsions were generated by the 10X Genomics Chromium Controller in the Northwestern Metabolomics Core Facility. Barcoded libraries were sequenced on the Illumina HiSeq 4000 platform. Raw sequence data were processed using the 10X Genomics Cell Ranger 3.1.0 pipeline for demultiplexing, trimming, aligning, and mapping to genes. After filtering of the scRNA-Seq data, we detected 5,411 cells with 54,770 reads per cell from WT control eyes; 10,806 cells with 27,239 reads per cell from WT lasered eyes; 10,584 cells with 27,329 reads per cell from *Ccr2^–/–^* control; and 7,414 cells with 38,135 reads per cell from *Ccr2^–/–^* lasered eyes. Sequencing files were deposited on GEO (GSE222094).

### Bioinformatics.

The filtered_feature_bc_matrix output files from Cell Ranger were loaded into Seurat v3 (23, 53). The 4 samples were merged using the “merge” function because they were sequenced together. Cells were filtered to include gene counts > 200 and < 5,000 features and < 8% mitochondrial DNA. We next performed data normalization (NormalizeData, LogNormalize, scale factor = 10,000), found variable features (method = vst, 2,000 features), ran ScaleData, and performed RunPCA (npcs = 50) on the merged object. An elbow plot was used to determine the number of principal components to include. Cells were clustered using FindNeighbors (dimensions = 1:18) and FindClusters (resolution = 0.4) and were visualized by RunUMAP. Cells were annotated by known marker genes including Mg (*Tmem119*, *P2ry12*, *Siglech*), Macs (*Fcgr1*, *C1qa*, *Aif1*), monocytes (*Spn*, *Ccr2*, *Ly6c2*), DCs (*Flt3*, *Zbtb46*), PMNs (*Csf3r*, *Cxcr2)*, B cells (*Cd79a*, *Igkc*), T cells (*Cd3d*, *Cd8b1*), NK cells (*Nkg7*, *Klre1*), proliferating cells (*Mki67*, *Top2a*), and contaminating retinal cells (*Clu*, *Rho*, *Rpe65*). Doublets were removed that expressed canonical markers from multiple cell types and contained greater than expected numbers of genes for cell type. For example, B cells and T cells each expressed ~1,500 genes, and the B cell–T cell doublet cluster expressed ~3,000 genes. After doublet removal, cells were rescaled and RunPCA was redone. Cells were again clustered using FindNeighbors (dimension = 1:18) and FindClusters (resolution = 0.4) and were visualized by RunUMAP. The subset function was used to subset mononuclear phagocytes, and the above process was repeated to recluster mononuclear phagocytes time (dimensions = 1:18, resolution = 0.4). The subset function was again used to subset Macs, and the above process was repeated a final time (dimensions = 1:10, resolution = 0.6).

Hypergeometric enrichment analysis was performed using the phyper function (*P* < 0.001). Differential expression analysis was performed using FindAllMarkers (min.pct = 0.25, fold change = 2; Supplemental Table 4). GO enrichment analysis was performed on upregulated genes > 2-fold with adjusted *P* <0.001. Gorilla was used for GO enrichment (32), using a background of genes expressed in > 25% of Macs. Selected GO enrichment terms are displayed in Figure 4A; all GO enrichment terms can be found in Supplemental Table 5. The DotPlot function was used to visualize genes that were included in selected GO terms. Specific code is available upon request.

### IF imaging of choroidal whole mounts.

Enucleated eyes were stained as described (51). Briefly, choroidal whole mounts (choroid/sclera/RPE) were fixed and stained with the antibodies described in Table 1. Images were obtained on a Nikon W1 Dual Cam Spinning disk confocal microscope in the Northwestern Center for Advanced Microscopy using Nikon NIS Elements software (Nikon). Images were masked, and ImageJ software (NIH) was used to measure CNV area.

### Multiparameter flow cytometry analysis.

Eyes were enucleated directly into HBSS. Eyes were dissected to isolate retina and choroid/RPE/sclera complex. Retina and choroid/RPE/sclera complex were cut into 4 pieces and digested in Liberase TL for 1 hour at 37°C with shaking (200 rpm). After digestion, single-cell suspensions were treated identically to our previously published flow cytometry procedure (22). The innate immune cell marker antibodies can be found in Table 1. Samples were run on a FACSymphony A5-Laser Analyzer (Becton Dickinson), and data were analyzed using FlowJo version 10 (FlowJo).

### Statistics.

Comparisons of mononuclear phagocyte numbers between WT and lasered mice were performed using unpaired or Welch’s 2-tailed *t* test based upon differences in variances between groups. Mac and DC numbers between control, PBS + laser–treated mice, and DT + laser–treated mice were made using Brown-Forsythe and Welch’s 1-way ANOVA followed by Dunnett’s T3 multiple comparisons test because variances between groups were not equal. Laser CNV area normality was tested using the Shapiro-Wilk test. Since the data were not normally distributed, the Mann Whitney *U* test was used to compare PBS- and DT-treated mice. *P* < 0.05 was considered significant. Data represent mean ± SEM in Figure 6, Figure 7, and Figure 8.

### Study approval.

All studies were performed in accordance with protocols approved by the Northwestern University IACUC.

## Author contributions

SD’s roles included data curation, investigation, methodology, and writing of the original draft. AR’s role was investigation, methodology, and writing, reviewing, and editing the manuscript. CMC’s roles were formal analysis, methodology, and writing, reviewing, and editing the manuscript. HP’s roles were conceptualization and writing, reviewing, and editing the manuscript. JAL’s roles were conceptualization, data curation, formal analysis, funding acquisition, investigation, methodology, project administration, supervision, visualization, writing the original draft, and editing and reviewing the manuscript.

## Supplementary Material

Supplemental table 1

Supplemental table 2

Supplemental table 3

Supplemental table 4

Supplemental table 5

## Figures and Tables

**Table 1:**
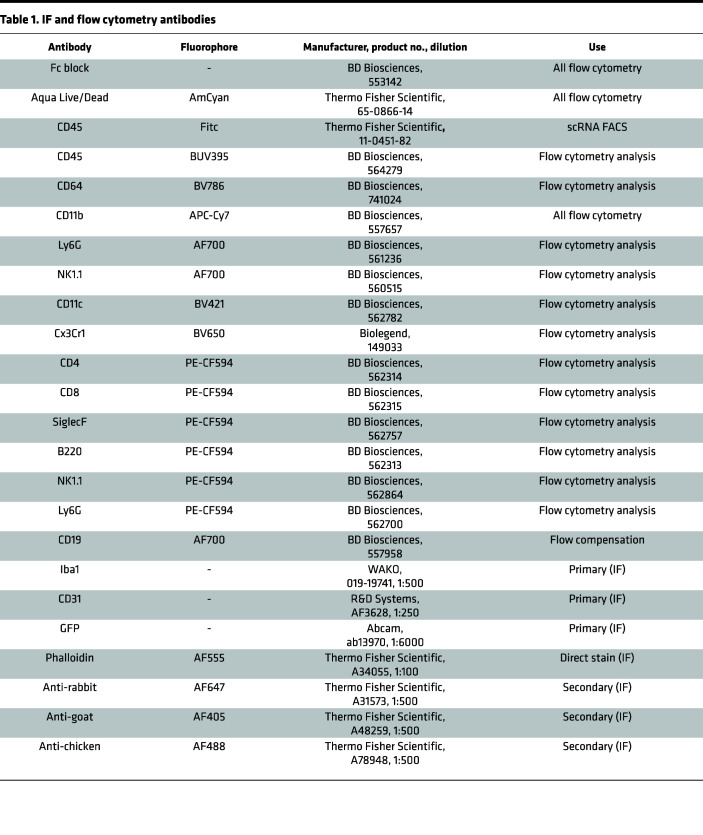
IF and flow cytometry antibodies

## Version 1 02/23/2023

In-Press Preview

## Version 2 04/10/2023

Electronic publication
