# Supplementary material for: CD11c+ macrophages are proangiogenic and necessary for experimental choroidal neovascularization
Source: JCI Insight. 2023 Apr 10;8(7):e168142. doi: 10.1172/jci.insight.168142 (PMC10132149; doi:10.1172/jci.insight.168142)
Supplement: Supplemental table 1 [file jciinsight-8-168142-s169.pdf]

Supplemental Table 1. Proportion of cells from each cluster as a function of experimental group from all $CD45^{+}$ cells (Figure 1).

	WT Control	WT Laser	*Ccr2*$^{-/-}$ Control	*Ccr2*$^{-/-}$ Laser
Mg1	0.2090	0.1238	0.2285	0.1610
Mg2	0.0298	0.0124	0.0391	0.0161
Mg3	0.0130	0.0146	0.0077	0.0101
Mac1	0.1676	0.0605	0.1569	0.0771
Mac2	0.0198	0.1702	0.0039	0.0164
Mac3	0.0163	0.0105	0.0107	0.0089
Mac4	0.0062	0.0070	0.0060	0.0032
Monocyte	0.0978	0.0878	0.0726	0.0538
DC1	0.0436	0.0723	0.0307	0.0285
DC2	0.0017	0.0109	0.0012	0.0050
PMN1	0.0267	0.0298	0.0334	0.2365
PMN2	0.0014	0.1057	0.0008	0.1309
B cell	0.1643	0.1090	0.1943	0.0797
T1	0.0624	0.0339	0.0582	0.0273
T2	0.0304	0.0409	0.0373	0.0283
NK	0.0864	0.0779	0.0950	0.0717
KI67	0.0107	0.0218	0.0096	0.0295
Retina	0.0076	0.0032	0.0054	0.0044
Doublet	0.0052	0.0077	0.0086	0.0117
