# Supplementary material for: CD11c+ macrophages are proangiogenic and necessary for experimental choroidal neovascularization
Source: JCI Insight. 2023 Apr 10;8(7):e168142. doi: 10.1172/jci.insight.168142 (PMC10132149; doi:10.1172/jci.insight.168142)
Supplement: Supplemental table 2 [file jciinsight-8-168142-s170.pdf]

Supplemental Table 2. Proportion of cells from each cluster as a function of experimental group from all mononuclear phagocytes cells (Figure 2).

	WT Control	WT Laser	*Ccr2*$^{-/-}$ Control	*Ccr2*$^{-/-}$ Laser
Mg-A	0.2977	0.0515	0.3579	0.1630
Mg-B	0.0420	0.1530	0.0455	0.2475
Mg-C	0.0533	0.0300	0.0761	0.0574
Mg-D	0.0226	0.0272	0.0162	0.0347
Mg-E	0.0116	0.0101	0.0078	0.0100
Mac-A	0.1637	0.0547	0.1659	0.1196
Mac-B	0.1063	0.0450	0.0977	0.0721
Mac-C	0.0113	0.0144	0.0110	0.0132
C-MDM	0.0325	0.2850	0.0071	0.0482
CMo	0.0567	0.0605	0.0207	0.0315
NCMo-1	0.1138	0.0949	0.1098	0.1036
NCMo-2	0.0027	0.0016	0.0054	0.0020
cDC-1	0.0137	0.0197	0.0140	0.0104
cDC-2	0.0554	0.0974	0.0362	0.0578
migDC	0.0024	0.0194	0.0019	0.0120
pDC	0.0014	0.0035	0.0004	0.0060
B Cell	0.0089	0.0168	0.0179	0.0052
T Cell	0.0041	0.0154	0.0086	0.0060
