# Supplementary material for: CD11c+ macrophages are proangiogenic and necessary for experimental choroidal neovascularization
Source: JCI Insight. 2023 Apr 10;8(7):e168142. doi: 10.1172/jci.insight.168142 (PMC10132149; doi:10.1172/jci.insight.168142)
Supplement: Supplemental table 3 [file jciinsight-8-168142-s171.pdf]

Supplemental Table 3. Proportion of cells from each cluster as a function of experimental group from all macrophages (Figure 3).

	WT Control	WT Laser	*Ccr2*$^{-/-}$ Control	*Ccr2*$^{-/-}$ Laser
Resting Mg 1	0.365	0.034	0.401	0.083
Resting Mg 2	0.075	0.046	0.097	0.080
Lasered Mg 1	0.069	0.232	0.099	0.422
Lasered Mg 2	0.028	0.058	0.015	0.035
Glycoltic Mg	0.017	0.017	0.010	0.014
IFN Mg	0.009	0.010	0.007	0.015
Lyve1+ Mac	0.190	0.072	0.185	0.139
MHCII+ Mac	0.139	0.071	0.125	0.091
Mrc1+ Mac	0.049	0.015	0.037	0.040
Folr2+ Mac	0.015	0.020	0.014	0.011
Ccr2+ MDM	0.029	0.287	0.004	0.024
Spp1+ MDM	0.010	0.128	0.002	0.027
PMN	0.005	0.010	0.003	0.020
